# Visual Hallucinations in First-Episode Psychosis: Association with Childhood Trauma

**DOI:** 10.1371/journal.pone.0153458

**Published:** 2016-05-04

**Authors:** Martine Solesvik, Inge Joa, Tor Ketil Larsen, Johannes Langeveld, Jan Olav Johannessen, Jone Bjørnestad, Liss Gøril Anda, Jens Gisselgård, Wenche ten Velden Hegelstad, Kolbjørn Brønnick

**Affiliations:** 1 TIPS-Centre for Clinical Research in Psychosis, Stavanger University Hospital, Stavanger, Rogaland, Norway; 2 Network for medical sciences, University of Stavanger, Stavanger, Rogaland, Norway; Chiba University Center for Forensic Mental Health, JAPAN

## Abstract

**Background:**

Hallucinations are a core diagnostic criterion for psychotic disorders and have been investigated with regard to its association with childhood trauma in first-episode psychosis samples. Research has largely focused on auditory hallucinations, while specific investigations of visual hallucinations in first-episode psychosis remain scarce.

**Objectives:**

The aims of this study were to describe the prevalence of visual hallucinations, and to explore the association between visual hallucination and childhood trauma in a first-episode psychosis sample.

**Methods:**

Subjects were included from TIPS-2, a first episode psychosis study in south Rogaland, Norway. Based on the medical journal descriptions of the Positive and Negative Symptoms Scale (PANSS), a separate score for visual and auditory hallucinations was created (N = 204). Patients were grouped according to hallucination severity (none, mild, and psychotic hallucinations) and multinomial logistic regression was performed to identify factors associated with visual hallucination group.

**Results:**

Visual hallucinations of a psychotic nature were reported by 26.5% of patients. The experience of childhood interpersonal trauma increased the likelihood of having psychotic visual hallucinations.

**Conclusion:**

Visual hallucinations are common in first-episode psychosis, and are related to childhood interpersonal trauma.

## Introduction

Hallucinations are a major group of clinical symptoms with diagnostic significance in the psychosis spectrum disorders [1], and in first-episode psychosis (FEP) the majority of patients report hallucinatory experiences [2, 3]. Hallucinations may be defined as perceptual experiences in the absence of external stimuli, and can involve several sensory modalities, such as auditory, visual, olfactory and tactile [4, 5]. Auditory hallucinations are the most common, with commenting and commanding voices frequently reported [2]. Voices arguing and commenting were named “first rank” symptoms by Schneider, and were included in the DSM-IV as sufficient symptomatic criteria for a schizophrenia diagnosis. This special status of first rank symptoms has been removed in DSM-5, granting equal importance to hallucinations in any modality in diagnosing psychosis.

Visual hallucinations have often been linked to biological causes, such as organic disease and drug-induced psychosis, and have been considered uncommon in psychiatric samples [6]. However, a work group for the Consortium on Hallucination Research (ICHR) recently concluded that visual hallucinations are relatively common in psychosis [7]. Further, a review by Waters and colleagues reported prevalence rates of 27% among patients with schizophrenia [6]. Similarly, in a FEP sample (N = 143), 21% reported having visual hallucinations [2]. In spite of this, while auditory hallucinations have been extensively investigated [8], research focusing on visual hallucinations remains relatively scarce.

Although termed “non-organic”, psychotic illness has traditionally been understood within a biomedical framework, where biology and genetics have been prominent explanatory factors [9]. More recent aetiological models of psychosis have included environmental factors, assuming that psychological processes play a part in both the development and maintenance of psychotic symptoms [10–12]. One of the major theoretical approaches in this direction is the traumagenic neurodevelopmental model, introduced in 2001 [11]. This model proposes that childhood trauma may cause brain abnormalities and increased sensitivity to stress. This trauma-induced vulnerability may affect individuals with and without a genetic predisposition to stress sensitivity into adulthood, and may increase their sensitivity to later stressors, which in turn may contribute to the development of psychosis. A sound body of clinical research currently supports this model [13].

Trauma in the form of childhood abuse has been implicated in psychosis development [14–16], and an association between childhood trauma and specific symptoms of psychosis, including hallucinations, has been demonstrated [10, 17–20]. Childhood interpersonal trauma has also been found to increase the likelihood of experiencing visual hallucinations in much larger non-clinical samples [18, 21]. Although an association between childhood trauma and visual hallucinations has been suggested [10, 18, 19, 22, 23], studies tend to not differentiate between modalities of hallucinatory experiences or to focus mainly on the auditory modality. However, in a study of patients with first-episode schizophrenia (N = 57) by 00DCçok and colleagues, the association between both visual and auditory hallucinations and childhood trauma were investigated, with childhood emotional abuse and childhood physical neglect being related to the severity of visual hallucinations [24], but severity of auditory hallucinations was not adjusted for. The present study is a replication of this study, but in a FEP sample and with multivariate analyses controlling for confounding variables such as auditory hallucinations.

This study aims first, to describe the prevalence and simple phenomenology of visual hallucinations in a FEP sample. Second, the aim is to investigate their association with childhood interpersonal trauma. Based on the literature, we expected the experience of childhood trauma to be associated with an increased probability of experiencing visual hallucinations.

## Methods

### Sample and recruitment

The sample was recruited from the on-going TIPS-2 study (early Treatment and Intervention in Psychosis), a naturalistic follow-along FEP study in south-Rogaland, Norway, including individuals with FEP between 2002–2014. All participants provided written informed consent. The project was approved by the Regional Committee for Medical Research Ethics Health Region West, Norway (015.03). Detailed descriptions of the inclusion criteria and methods have been published elsewhere [25, 26].

Patients who were included in the study met the following criteria: living in the catchment area (Rogaland county); age 15–65 years; meeting the DSM-IV criteria for a first episode of schizophrenia, schizophreniform psychosis, schizoaffective psychosis, delusional disorder, brief psychosis, affective disorder with mood incongruent delusions, or psychosis not otherwise specified, and also from August 1, 2008 substance induced psychosis (excluded for the purpose of the present study); being actively psychotic as measured by the Positive and Negative Syndrome Scale (PANSS)[27]; not previously receiving adequate treatment of psychosis; no neurological or endocrine disorders related to the psychosis; living in the catchment area, understands and speaks one of the Scandinavian languages; an IQ over 70; and being able and willing to sign an informed consent. The patients agreed to baseline assessment, and follow-up after 3 months, and 1, 2 and 5 years. This study is based on data from the baseline assessment. Of the 482 individuals identified, 70 of them were excluded because they failed to meet the inclusion criteria. 165 of the remaining 412 individuals refused to participate. There were 14 individuals for whom the trauma baseline data was missing and a full PANSS score was missing for 29 individuals, leaving a total of 204 first-episode psychosis patients in this study.

### Clinical Measures

PANSS [27] was used to assess severity of positive and negative symptoms of psychosis. In general, PANSS has been found to have good reliability and validity [28, 29], and the hallucinations (P3) item in PANSS has displayed a high ability to discriminate between symptom severity levels [30]. In addition to using the original P3 score, separate P3 scores for visual and auditory hallucinations were generated for all participants. DUP was defined as the time from onset of psychosis until the start of adequate treatment. Onset of psychosis was equated with the first appearance of positive psychotic symptoms, defined as the first week with symptoms corresponding to a PANSS score of 4 or more on positive subscale items 1, 3, 5 or 6 or on general subscale item 9. The Structured Clinical Interview for the DSM-IV Axis 1 Disorders (SCID) [31] was used for diagnostic purposes. The variable "core schizophrenia" indicated whether the patient met the A-criteria in DSM-IV for schizophrenia, such as in schizophreniform disorder, schizoaffective disorder or schizophrenia. Misuse of alcohol and other drugs was measured by the Drake scale [32]. Global functioning was measured using the Global Assessment of Functioning Scale (GAF) [33] divided into functioning (GAF-f) and symptom (GAF-s) subscales. The Premorbid Adjustment Scale (PAS) was used to measure premorbid functioning [34]. PAS covers two areas of functioning, school adaption and socialization, described through initial childhood and adolescence. Brief Betrayal Trauma Survey (BBTS) [35], a self-report measure to identify traumatic experiences both in childhood and adulthood was employed. The BBTS inquires about whether participants have experienced any of four types of traumatic events: non-interpersonal trauma; interpersonal trauma by someone not close to them; interpersonal trauma perpetrated by someone close to them; and other trauma. The BBTS has been demonstrated to be a reliable measure [35].

### Assessment Reliability

The assessment personnel had previously been trained to reliable use of the assessment instruments, with inter-rater reliability measures ranging from fair to very good [25].

### Data Analysis

The prevalence of visual hallucinations was scored using baseline PANSS P3. All involved personell had provided extensive written narratives for each PANSS-interview, and the narratives in the original files were re-examined in order to rescore the hallucination variable. Auditory and visual hallucinations were given a separate P3 score based on the general hallucination criteria in PANSS. The reliability and validity of this procedure was evaluated by having a second rater scoring a subset of 23 randomly chosen patients. The two-way mixed intra-class correlation coefficient was 0.84, using a type-A coefficient assuming absolute agreement. This supports the reliability and validity of the scoring procedure. In the PANSS guidelines for scoring hallucinations a score of 1 indicates absence of hallucinatory experiences, whereas a score equal to or higher than 4 indicates hallucinatory experiences of a psychotic nature. Based on these cut off scores, patients were separated into three groups for the purpose of analysis: no visual hallucinations (P3 score = 1), mild visual hallucinations (P3 score = 2–3) and psychotic visual hallucinations (P3 score = 4–7). The same was done for auditory hallucinations scores.

Continuous variables were described as means and standard deviations, while categorical variables were analysed using cross-tabs. Prior to analyses, the prerequisites for multinomial logistic regression was evaluated by examining inter-correlations and collinearity statistics, following the approach recommended by Belsely, Kuh, and Welsch [36]. There were no indications of multicollinearity. Multinomial logistic regression was used to test the hypothesis that childhood trauma would increase the likelihood of experiencing visual hallucinations. The groups of no, mild and psychotic visual hallucinations were entered as the dependent variable, with no visual hallucinations as a reference group. Age, gender, “core schizophrenia”, childhood social and academic adjustment, childhood interpersonal and non-interpersonal trauma, the continuous score for auditory hallucinations, alcohol abuse, and drug abuse were entered as predictor variables. The same procedure was performed with the groups of no, mild and psychotic auditory hallucinations as the dependent variable, with no auditory hallucinations as reference group. All analyses were performed with SPSS, version 22.

## Results

### Demographic and Clinical Characteristics

Sample characteristics are described in Table 1. At baseline, average age was 26.6 years, 43.3% were female, 31.5% had experienced childhood interpersonal trauma, 16.3% had experienced childhood non-interpersonal trauma, and drug abuse was prevalent in 25.7% of the patients.

**Table 1:** Descriptive Statistics for Demographic and Clinical Characteristics, Premorbid adjustment and Childhood Trauma Across PANSS Derived Groups of Visual Hallucinations*.

	No VH (N = 88)	Mild VH (N = 61)	Psychotic VH (N = 54)	Total (N = 204)
**Demography**				
Age (years)	28.61 (11.25)	24.75 (8.75)	25.54 (10.02)	26.64 (10.33)
% Female (N)	36.4 (32)	47.5 (29)	53.7 (29)	44.3 (90)
**Clinical status**				
Age of onset	26.77 (11.36)	23.09 (7.75)	24.53 (10.36)	25.06 (10.44)
DUP median (I.Q. range)	12.50 (38.3)	17.50 (103.0)	13.50 (51.0)	13.50 (51.0)
No AH	48.9 (43)	17.7 (11)	14.8 (8)	30.4 (62)
% Core schizophrenia (N)	45.45 (40)	21.31 (13)	44.44 (24)	37.93 (77)
Mild AH	13.6 (12)	43.5 (27)	14.8 (8)	23 (47)
Psychotic AH	37.5 (33)	38.7 (24)	70.4 (38)	46.6 (95)
PANSS P3 AH	2.53 (1.67)	3.08 (1.26)	3.70 (1.41)	3.01 (1.56)
PANSS P3 VH	1.00 (.00)	2.82 (.39)	4.15 (.41)	2.39 (1.34)
PANSS negative	2.31 (1.09)	1.98 (0.77)	2.06 (1.02)	2.14 (0.99)
PANSS disorganized	2.04 (1.14)	1.95 (0.95)	2.02 (1.18)	2.01 (1.09)
PANSS depressive	3.06 (1.14)	3.28 (1.08)	3.44 (0.95)	3.23 (1.08)
PANSS positive	2.90 (0.80)	3.02 (0.90)	3.34 (0.87)	3.05 (0.86)
PANSS excitative	1.57 (0.73)	1.49 (0.65)	1.56 (0.63)	1.55 (0.68)
GAF symptoms	30.88 (7.79)	32.67 (6.51)	30.93 (7.52)	31.42 (7.37)
GAF functioning	38.68 (8.40)	41.02 (9.65)	40.78 (11.49)	39.94 (9.69)
% Drug abuse (N)	21.8 (19)	19.7 (12)	38.9 (21)	25.7 (52)
% Alcohol abuse (N)	10.2 (9)	13.1 (8)	16.7 (9)	12.8 (26)
% Remission 12 months (N)	44.7 (34)	42.6 (20)	46.5 (20)	44.6 (74)
**Premorbid adjustment**				
Social				
Child	0.93 (1.31)	1.05 (1.25)	1.23 (1.36)	1.04 (1.30)
Early adolescence	1.18 (1.16)	1.27 (1.23)	1.41 (1.20)	1.27 (1.19)
School				
Child	1.64 (1.18)	2.15 (1.38)	1.77 (1.29)	1.82 (1.28)
Early adolescence	2.05 (1.29)	2.53 (1.28)	2.76 (1.37)	2.38 (1.34)
**Childhood Trauma**				
% Interpersonal (N)	23.9 (21)	31.1 (19)	44.4 (24)	31.5 (64)
% Non-interpersonal (N)	18.2 (16)	11.5 (7)	18.5 (10)	16.3 (33)

* Data are reported as mean (SD) except where noted. AH: Auditory hallucination, VH: visual hallucinations, DUP: duration of untreated psychosis in weeks, I.Q. range: Interquartile range

### Prevalence of Visual Hallucinations

The prevalence of psychotic visual hallucinations was 26.5% (N = 54), 30.4% (N = 62) had mild visual hallucinations, while 43.1% (N = 88) had no visual hallucinations. The prevalence of psychotic auditory hallucinations was 39.6% (N = 95), while 19.6% (N = 47) had mild auditory hallucinations, and 25.8% (N = 62) had no auditory hallucinations.

### Simple Phenomenology of the Visual Hallucinations

In the PANSS interviews, the content of visual hallucinations was mainly reported to be either people, transient visual experiences (e.g. shadows, changing faces or objects), of religious nature (e.g. Jesus/God, angels, demons/devils), animals or visual patterns (e.g. stars, lights). They ranged from clear and detailed percept-like hallucinations to experiences of shadows in the visual periphery. Duration varied from seconds to over 30 minutes, while frequency ranged from one-time occurrence to daily presence. Visual hallucinations were most often experienced as frightening and anxiety inducing, but some experiences elicited a more neutral reaction or were even perceived as benevolent and protective. Some patients reported that they knew it was not real, whereas others believed firmly that their visual hallucinations represented reality. Some of these patients had delusions tied to the visual experience.

### Visual Hallucinations and childhood trauma

The hypothesized association between childhood trauma and visual hallucinations was investigated using multinomial logistic regression with visual hallucination group as dependent variable. The omnibus vs. intercept only model likelihood ratio test chi-square (66.86) indicated that the full model showed a significantly better fit to the data than did the intercept only model (p< .001). Table 2 shows the adjusted odds ratios for the odds of mild or psychotic visual hallucinations vs. the reference category of no visual hallucinations in the full model.

**Table 2:** Adjusted Odds Ratios for the Odds of Mild Visual Hallucinations or Psychotic Visual Hallucinations vs. No Visual Hallucinations for the Full Model.

	Mild Visual Hallucinations		Psychotic Visual Hallucinations	
	OR (95% CI)	P-value	OR (95% CI)	P-value
Age	0.96 (0.93–1.00)	.063	0.99 (0.95–1.03)	.493
Core schizophrenia	0.30 (0.13–0.69)	**.005**	0.81 (0.34–1.93)	.637
Childhood social adjustment	0.97 (0.70–1.33)	.830	1.14 (0.82–1.60)	.432
Childhood academic adjustment	1.46 (1.05–2.04)	**.024**	1.01 (0.70–1.45)	.971
Gender (female)	1.17 (0.52–2.62)	.700	2.46 (0.99–6.11)	.052
Child Interpersonal trauma	1.55 (0.66–3.65)	.317	3.02 (1.27–7.16)	**.012**
Child Non-interpersonal trauma	0.31 (0.09–1.03)	.086	0.70 (0.24–2.06)	.511
Alcohol abuse	2.05 (0.58–7.28)	.265	1.91 (0.54–6.77)	.317
Drug abuse	0.45 (0.16–1.31)	.143	2.29 (0.88–6.00)	.093
Auditory hallucinations	1.39 (1.08–1.79)	**.011**	1.78 (1.34–2.37)	**.000**

OR: Odds ratio; CI: Confidence interval. Alpha level 0.05. The bold values in the table indicate the variables that reached statistical significance. Reference group: no visual hallucinations

Interpersonal childhood trauma increased the odds of psychotic visual hallucinations by a factor of three. Gender showed a trend towards significance (p = .052), with females being twice as likely to have such visual experiences. Auditory hallucinations were positively associated with both mild and psychotic visual hallucinations in comparison with no visual hallucinations. The occurrence of childhood non-interpersonal trauma was less likely among those with mild visual hallucination in comparison to those with no visual hallucinations. Mild visual hallucinations were associated with poorer childhood academic adjustment, as indicated by higher PAS scores. Age trended towards significance (p = .063) and was negatively associated with mild visual hallucinations, i.e. such hallucinatory experiences were less likely to occur with increasing age in participants. Being diagnosed with “core schizophrenia” was associated with mild visual hallucinations, however it did not have an effect on psychotic visual hallucinations.

### Auditory Hallucinations and Childhood Trauma

A similar multinomial logistic regression was also performed with the trisected variable of auditory hallucinations as the dependent variable. The omnibus vs. intercept only model likelihood ratio test chi-square (35.115) indicated that the full model showed a significantly better fit to the data than the intercept only model (p = .009). Visual hallucinations were positively associated with both mild and psychotic auditory hallucinations when compared with no auditory hallucinations. None of the other predictor variables increased the likelihood of hallucinatory experiences in the auditory modality.

## Discussion

The main findings of this study were that FEP patients reporting childhood interpersonal trauma had increased likelihood of also reporting psychotic visual hallucinations. The prevalence of psychotic visual hallucinations was 26.5% (N = 54).

The prevalence of psychotic visual hallucinations in this FEP sample is similar to the mean prevalence rate for schizophrenia, reported to be 27% in a recent review [6]. This indicates that such experiences also are common in first-episode psychosis. Visual and auditory hallucinations tend to co-occur in this sample, with the presence of visual hallucinations increasing the probability of having auditory hallucinations, and vice versa, but visual hallucinations were nevertheless independently associated with childhood interpersonal trauma. However, some patients have visual hallucination without having experienced childhood trauma, and some experience childhood trauma and never develop visual hallucinations. Thus, childhood trauma is neither sufficient nor necessary to explain visual hallucinations by itself.

Childhood trauma is associated with a range of mental health problems, and is not specific to psychosis [13]. The traumagenic neurodevelopmental model explains how childhood trauma may influence psychological and neurobiological processes, leading to a broad spectrum of symptoms and disorders, including psychosis [11, 13]. The model proposes a dissociative response to childhood trauma to be a potential pathway to the positive symptoms of psychosis [11]. A dissociative tendency, measured using The Dissociative Experiences Scale (DES), has been found to mediate the association between childhood trauma and hallucinatory experiences [16, 37], and could be a potential explanatory factor for the association between childhood trauma and visual hallucinations in this study. Hypervigilance is common in individuals who have experienced traumatic events, and is commonly seen in post-traumatic stress disorder [38]. A recent model suggests that some hallucinations may be explained by the individuals' hypervigilance to threat stimuli [39]. This hypervigilance is explained as a bi-product of probabilistic biases of the perceptual system. While under stress or threat, an individual will be more likely to adjust perceptual bias to reduce the probability of false negatives, at the expense of an increased risk of accepting false positive perception [39].

Hypervigilant auditory hallucination has been proposed to be a distinct subtype of auditory hallucinations [39, 40], and a recent study suggests a false positive threat perception to be a possible mediating factor also in the visual domain [41]. Such hypervigilance could contribute to the understanding of the relationship between childhood interpersonal trauma and visual hallucinations. Confusion between imagination and sensory perception might also contribute to visual hallucinations [42]. Intrusive trauma-related images may lead to source monitoring errors, a documented effect of trauma [10].

A meta-analysis of 36 studies indicated that childhood trauma increase the risk of developing psychosis. Large non-clinical and clinical studies have found a dose-response relationship between childhood trauma and psychotic symptoms [23, 24, 37], with risk increasing by each exposure to an additional type of adverse event. No differentiation between types and numbers of interpersonal trauma was made in our analyses, limiting the ability to specify the association between trauma and visual hallucinations. Childhood rape, molestation and neglect [18], as well as bullying [10] have been found to increase the likelihood of experiencing visual hallucinations in non-clinical samples., whereas childhood emotional abuse and physical neglect have been found in a clinical sample [24]. Further investigation of the relationship between different types of interpersonal trauma and diverse modalities of hallucinations, as well as potential mediators, in clinical samples would be of interest in future studies. An exploration in a FEP sample would be particularly advantageous to avoid potential confounding factors present in chronic samples [43]. According to the traumagenic neurodevelopmental model, the effect of trauma may be reversible with psychotherapy [10]. This highlights the importance of clinician inquiry about trauma; if therapy is aimed at alleviating effects of trauma and reducing the patients stress sensitivity, this might in turn lower the probability of developing psychosis.

This study is based on data collected as part of the TIPS-2 study, which recruited FEP patients in a well-defined catchment area constituted of about 290 000 individuals. It is assumed that nearly all FEP patients in the designated area were identified [25]. In contrast with chronic psychosis samples, a FEP sample eliminates potential biases related to psychosis chronicity, such as effects of illness duration and severity, not being responsive to treatment, not being compliant with treatment, and the possible effect of prolonged use of antipsychotic medication [43].

### Limitations

The analyses in this paper were based on data from the TIPS-2 study, with no particular focus on visual hallucinations when data were collected. Some of the PANSS reports were more detailed in their description than others, limiting the value or feasibility of representative phenomenological descriptions. Data collected with the specific purpose of investigating visual hallucinations might have provided more in-depth information. Some question the reliability of retrospective self-report based trauma assessment in psychotic samples. According to a review of Read and colleagues, reports by patients with a psychotic illness tend to be reliable, if anything they tend to under-report rather than over-report [10]. Of patients meeting the inclusion criteria 40% refused to participate in the study, it is possible that this group differs from the participating group, and that the inclusion of these patients would have affected the results.

## Conclusions

Childhood trauma has been implicated in the aetiology of psychosis and hallucinations in general. This study also support findings from non-clinical samples and a smaller clinical sample, indicating that childhood interpersonal trauma increases the likelihood of experiencing visual hallucinations. More knowledge about visual hallucinations in general is needed, and a further exploration of the relationship between interpersonal trauma and visual hallucinations in psychiatric samples would be of value.

## Supporting Information

S1 File TIPS PloSOne english.sav. (SAV)
